# AuNPs Hybrid Black ZnO Nanorods Made by a Sol-Gel Method for Highly Sensitive Humidity Sensing

**DOI:** 10.3390/s18010218

**Published:** 2018-01-13

**Authors:** Hongyan Zhang, Min Zhang, Cunchong Lin, Jun Zhang

**Affiliations:** School of Physical Science and Technology, Xinjiang University, Urumqi 830046, China; zhanghongyanxj@163.com (H.Z.); minzhang0816@163.com (M.Z.); lccz123@163.com (C.L.)

**Keywords:** black ZnO, AuNPs hybrid ZnO, nanorods, humidity sensor

## Abstract

A highly sensitive self-powered humidity sensor has been realized from AuNPs hybrid black zinc oxide (ZnO) nanorods prepared through a sol-gel method. XRD pattern reveals that both ZnO and ZnO/AuNPs exhibit a wurtzite structure. ZnO/AuNPs nanorods grow in a vertical alignment, which possesses high uniformity and forms dense arrays with a smaller diameter than that of ZnO nanoparticles. All ZnO/AuNPs and pure black ZnO show lower band gap energy than the typically reported 3.34 eV of pure ZnO. Furthermore, the band gap of ZnO/AuNPs nanocomposites is effectively influenced by the amount of AuNPs. The humidity sensing tests clearly prove that all the ZnO/AuNPs humidity sensors exhibit much higher response than that of ZnO sensors, and the sensitivity of such ZnO/AuNPs nanorods (6 mL AuNPs) display a change three orders higher than that of pure ZnO with relative humidity (RH) ranging from 11% to 95% at room temperature. The response and recovery time of the ZnO/AuNPs are 5.6 s and 32.4 s, respectively. This study of the construction of semiconductor/noble metal sensors provides a rational way to control the morphology of semiconductor nanomaterials and to design a humidity sensor with high performance.

## 1. Introduction

Humidity sensors for detecting relative humidity (RH) are very important in many fields of technology and our daily life, such as industrial production, public transportation, health care and food storage [1,2,3]. In terms of different conduction principles, the multiple types of traditional RH sensors have been developed, including electrical capacitance, optical and resistance sensors [4,5,6]. Recently, resistive-type humidity sensors have received considerable attention due to their high sensitivity, good linearity, operating stability and direct current electrical conductivity. Resistive-type humidity sensors make frequent use of the main categories of humidity sensing materials, which are ceramics, carbon materials, polymers, and metal oxide semiconductors [7,8,9]. Among these materials, metal oxide semiconductors are the most common material type used for humidity sensors on account of their ease of fabrication, and controllable size and morphology. Of the many metal oxide semiconductor materials, ZnO has attracted considerable attention for use in the fabrication of RH sensors. ZnO is an n-type II-VI functional semiconductor material having a wide band gap (3.37 eV) hexagonal wurtzite structure with unique electrical and optical properties such as high electron Hall mobility, biocompatibility, high transparency and favorable morphology [10,11]. Humidity sensors based on ZnO materials have been fabricated in various forms, such as single crystals, thin film and sintered pellets [12,13].

For traditional resistive-type ZnO-based humidity sensors, the water molecules get adsorbed on the surface of ZnO in molecular and hydroxyl form and cause electrical conduction at room temperature. In order to improve the sensing performance of ZnO, significant efforts have been invested to enhance the surface area of ZnO, thereby creating more active sites for rapid adsorption/desorption of water molecules, which directly contributes to improving the RH sensing properties [14]. Doping with a proper element is a very effective way to enhance the sensing performance. Furthermore, investigations have shown that the structures of ZnO strongly affect the physical and chemical properties and play an important role in the sensing properties. Recently, ordered structured ZnO nanorods have received much attention due to their large length-to-diameter, high surface-to-volume ratio and high electron mobility, and they have been extensively investigated in terms of highly sensitive humidity sensors [15,16]. Moreover, such a structure provides a direct pathway for charge transport along the axis of ZnO arrays. As a result, electron-hole pair recombination possibilities are considerably reduced. Many studies have controlled the size of ZnO nanorods by doping [17,18]. Au nanoparticles (AuNPs) are typical noble metal particles with unique physical and chemical properties. In particular, Au-semiconductor hybrids have been used to enhance the sensitivity, selectivity and response time performance of ZnO humidity sensors [19]. However, controlling ZnO nanorods by forming hybrid AuNPs has not been widely studied yet.

In this paper, a high performance humidity sensor based on AuNPs hybrid black ZnO nanorods has been fabricated by a sol-gel method. The sensitivity for humidity sensing of the AuNPs hybrid ZnO nanorods is much higher than that of pure black ZnO nanoparticles. Such a high performance improvement can be attributed to the high surface-to-volume ratio of ZnO/AuNPs nanorods and the surface plasmon (SPR) effect of AuNPs. This means AuNPs hybrid ZnO can probably lead to the adsorption of a large amount of water molecules on the surface of ZnO nanocomposites. Furthermore, the band gap of pure black ZnO with 3.07 eV can be effectively narrowed by the amount of AuNPs, which offers unique physicochemical and electronic properties for humidity sensors. To the best of our knowledge, humidity sensors based on ZnO/AuNPs nanorods have been rarely reported. The present results demonstrate a feasible approach to fabricate semiconductor humidity sensors with high performance.

## 2. Materials and Methods

### 2.1. Materials

Zinc acetate (Zn(CH_3_COO)_2_·2H_2_O), monoethanolamine (MEA) and chloroauric acid (HAuCl_4_·4H_2_O_2_, 48–50% Au basis) were purchased from Macklin (Shanghai, China, www.labgogo.com). All the reagents in this work were of analytical grade and used without any further purification. Deionized water was used throughout the study.

### 2.2. Apparatus

X-ray diffraction measurements (XRD) were performed on a D8 Advance diffractometer with Cu Kα radiation λ = 0.154 nm (Bruker, Karlsruhe, Germany). The transmission electron microscopy (TEM) images were obtained using a JEM-2100F transmission electron microscope (Hitachi, Tokyo, Japan). Scanning electron micrographs (SEM) were obtained from an S-4800 scanning electron microscope (Hitachi). Absorption spectra were acquired on a Lambda 650 UV-vis spectrophotometer (PerkinElmer, New York, US) with the wavelength ranging from 200.00 to 1000.00 nm. The impedance of humidity sensors were measured by Zennium workstation (Zahner, Kronach, Germany).

### 2.3. Synthesis of AuNPs

The obtained spherical AuNPs with an average diameter of 15 ± 2 nm were prepared by the citrate reduction method, which can be referred to our previous work [20]. Briefly, 20 mL of 0.5 mM HAuCl_4_ solution was heated up to 90 °C under vigorous stirring for 5 min. Then 20 mL of 0.25 mM sodium citrate solution was quickly added to the boiling solution with vigorous stirring. The color of the pale yellow solution changed from faintly gray to claret-red for another 30 min. Then, the solution was cooled down to room temperature and stored in a refrigerator 4 °C for later use.

### 2.4. Synthesis of ZnO/AuNPs Nanorods

To produce ZnO/AuNPs nanocomposites, different amounts of AuNPs were added into precursors. The synthesis precursors of ZnO/AuNPs were prepared by dissolving 2.3 g of Zn(CH_3_COO)_2_·2H_2_O in 30 mL different precursor solvents to ZnO/AuNPs (Table 1). The mixture solution was kept under vigorous stirring at 65 °C for 10 min. Then 1 mL MEA was added drop by drop into the mixture solution. The molar ratio of MEA to Zn(CH_3_COO)_2_·2H_2_O was 1:1 and the concentration of Zn(CH_3_COO)_2_·2H_2_O was maintained at 0.35 M. The whole mixture solution was continuously stirred for 2 h with PH being maintained at 7.2, then a turbid purple homogeneous sol of Au/ZnO was obtained. After being aged for 36 h, the resulting sol was heat-treated in a quartz-tube furnace at 600 °C for 2 h in nitrogen (N_2_).

### 2.5. Preparation of Humidity Sensors

**Figure 1:**
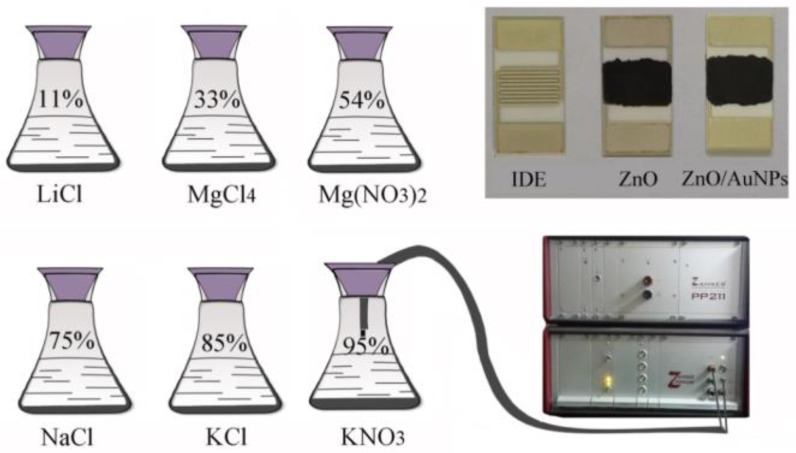
Schematic of the humidity sensing measurements.

To study the relative humidity response of the as-synthesized ZnO/AuNPs nanocomposites, the synthesized sample was mixed with DI water in a weight ratio of 5:1 to form a paste that was coated on the top of a five pair Ag-Pd interdigital electrodes (IDE) ceramic substrate (40 mm × 70 mm) to form a sensitive film. Then the film was dried in vacuum for 10 min at 60 °C and aged for 48 h at RT before RH experiments. The experimental humidity measurements and the photographs of actual humidity sensors based on both ZnO and ZnO/AuNPs are shown in Figure 1. In humidity sensing experiments, a controlled humidity environment was achieved by using supersaturated aqueous solutions of LiCl, MgCl_4_, Mg(NO_3_)_2_, NaCl, KCl and KNO_3_ which generate 11%, 33%, 54%, 75%, 85% and 95% RH at room temperature (25 °C), respectively. Two conducting wires with a portion firmly sealed in the rubber seal were connected between the sensing device and an external power supply. The response of the ZnO/AuNPs humidity sensors according to the change in RH was measured by the Zennium workstation at room temperature. The voltage was set to be AC 1 V and the frequency was changed from 40 Hz to 100 kHz in humidity studies. For the sensing response transient measurement, the sensing device was kept in the flask with 11 RH% at first. After the sensing response reached a steady state, the sensing device was then switched to another flask with different RH%. While exchanging the sensing device among flasks, the average switching time was shorted within 2 s to minimize possible interference with the response transients. Comparing ZnO with the ZnO/AuNPs nanocomposites, it is noticed that ZnO/AuNPs nanocomposites become darker and more metallic after being milled, which is attributed from the decreasing of the nanocrystals in diameter and the changes of the refractive index of the samples.

## 3. Results and Discussion

**Figure 2:**
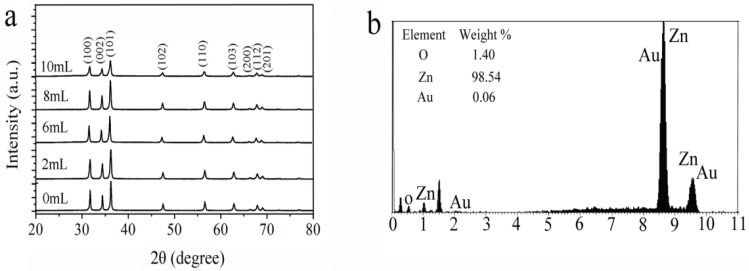
(**a**) XRD analysis of ZnO/AuNPs nanorods synthesized with different ratio of AuNPs; (**b**) EDS spectrum of the ZnO/AuNPs nanorods (Sample 3).

XRD analysis was used to investigate phase structure and crystalline size of ZnO/AuNPs with different amount of AuNPs. In Figure 2a, all the diffraction peaks present a good match with typical hexagonal wurtzite structure of ZnO (JCPDS card No. 80-0075), and these XRD data suggest that the obtained ZnO nanocomposites be of well crystallized hexagonal wurtzite type. We can observe that the intensity of the XRD peaks of pure ZnO is relatively high. For AuNPs hybrid ZnO nanocomposites with different amount of AuNPs, the XRD spectra show similar peaks related to that of pure ZnO and there is no additional XRD peak of AuNPs can be observed due to small amount of AuNPs. The intensities of (100), (002) and (101) diffraction peaks decrease with the increase of AuNPs amount. This result indicates that the crystallity of ZnO decreases when the amount of AuNPs is increased. Furthermore, no shifts in position of diffraction peaks are observed, indicating the formation of ZnO/AuNPs nanocomposites rather than the substitution of Au into ZnO crystal lattice or Au interstitial atom [21]. The chemical composition of ZnO/AuNPs nanocomposites is identified by energy dispersive X-ray (EDS) spectrum and the respective elemental concentrations are shown in Figure 2b. It reveals that the atomic percentage of Au is about 0.06%, the percentage of Zn is about 98.54% and the percentage of O is about 1.4%.

**Figure 3:**
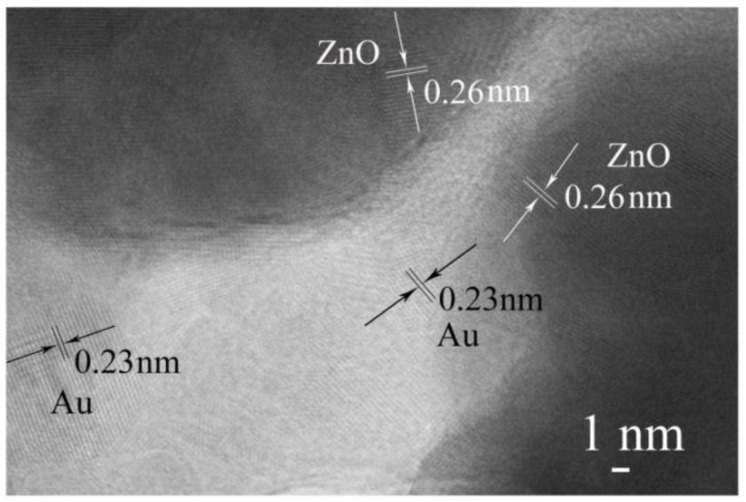
TEM images of hybrid ZnO/AuNPs nanorods (Sample 3).

Figure 3 shows the TEM images of ZnO/AuNPs nanorods (Sample 3). A magnified crystal structure is displayed in Figure 3 in which the lattice fringe of 0.26 nm can be indexed to the (002) plane of wurtzite structure of ZnO and the lattice fringe of 0.23 nm can be indexed to the (111) plane of face-centered-cubic Au [22]. The actual chemical composition of each sample determined by XRD is consistent with the EDS results and is found to be quite close to the target composition, which indicates the formation of AuNPs hybrid ZnO proceeds very well.

**Figure 4:**
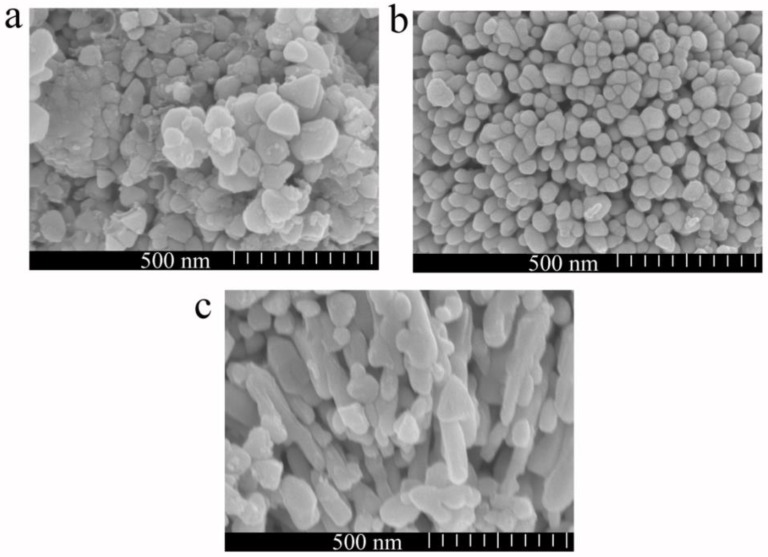
SEM surface image of (**a**) ZnO and (**b**) ZnO/AuNPs nanorods (Sample 3), (**c**) corresponding cross-section image of ZnO/AuNPs nanorods (Sample 3).

In order to measure the morphology of ZnO/AuNPs nanocomposites, scanning electron microscopy (SEM) observations were carried out as shown in Figure 4. The morphology image of the pure ZnO nanoparticles shows irregular disk-like nanostructures with diameters ranging from 60 nm to 200 nm in Figure 4a. ZnO/AuNPs nanorods show high uniformity and dense arrays with a smaller diameter from 45 nm to 60 nm as seen in Figure 4b. A cross-sectional SEM image of ZnO/AuNPs nanorods is shown in Figure 4c. The image shows that ZnO/AuNPs nanorods grow in a vertical alignment with a length from 200 nm to 300 nm. The morphology and structure of ZnO/AuNPs nanocomposites depended significantly on the AuNPs hybrids. Compared to pure ZnO nanoparticles, ZnO/AuNPs nanorods have a smaller particle size and more uniform morphology, which provides much larger length-to-diameter and surface-to-volume ratios than that of pure ZnO.

Some literature has reported that the size of black ZnO is nanoscale and the performance is superior to that of conventional white ZnO [23,24]. In this paper, SEM shows that the diameter size is from 60 nm to 200 nm, which confirms that it is black ZnO. Doping is more likely to affect the morphology and the structure of black ZnO, which will affect the humidity sensing performance.

**Figure 5:**
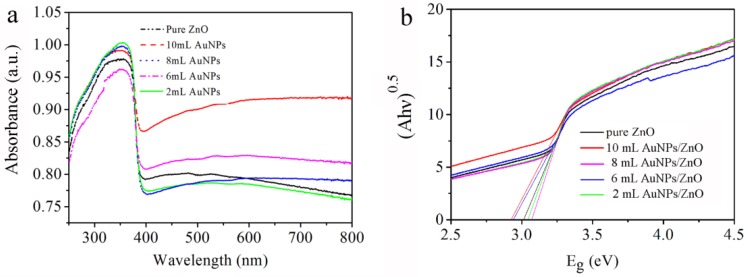
(**a**) Absorbance spectra and (**b**) Plot to determine the band gap energy of the AuNPs hybrid ZnO with different amount of AuNPs.

Figure 5 shows the UV-vis diffuse reflectance spectra and the derivative Kubelka-Munk functions of ZnO/AuNPs nanorods with different amounts of AuNPs. The absorption band peak at around 350 nm corresponds to the band gap absorption of ZnO. Compared with ZnO, the AuNPs hybrid ZnO nanorods display an obvious additional broader absorption band in the visible light region ranging from 400 nm to 800 nm. Considering the pure Au exhibits a sharp absorption peak centred at around 550 nm attributed to surface plasmon resonance (SPR) absorption of AuNPs [20], the board absorption bands in visible light of ZnO/AuNPs nanocomposites could be largely attributed to the aggregation of primary AuNPs hybrid ZnO. The surrounding environment of ZnO has been changed by the AuNPs hybrid ZnO nanorods, which affects the dielectric constant of ZnO/AuNPs nanorods. The Kubelka-Munk (F(R)) formula is used to determine the band gap energy of ZnO and ZnO/AuNPs nanocomposites. F(R) can be derived from the relation F(R) = (1 − R)^2^/2R = K/S, where R is the measured reflectance, K is the absorption and S is scattering coefficient. In the present work, optical band gaps of AuNPs hybrid ZnO from the precursor with different volumes of AuNPs of 0 mL, 2 mL, 6 mL, 8 mL and 10 mL are 3.01 eV, 3.04 eV, 2.94 eV, 3.06 eV and 2.92 eV respectively. The results reveal that all the ZnO/AuNPs and pure black ZnO show lower band gap energy compared to the typically reported 3.34 eV of pure ZnO [10,11]. Furthermore, the band gap of ZnO/AuNPs nanocomposites is effectively influenced by the amount of AuNPs.

**Figure 6:**
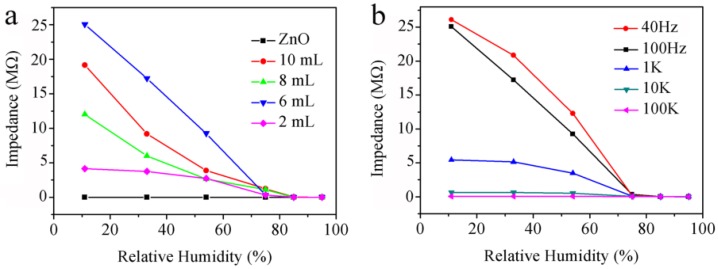
(**a**) Humidity sensing curves showing the decrease in impedance with the increase of the relative humidity for ZnO and ZnO/AuNPs with different amounts of AuNPs; (**b**) Relationship of impedance and relative humidity based on ZnO/AuNPs nanorods (Sample 3) at various frequencies.

Figure 6a shows the change in conductivity of ZnO/AuNPs sensors as a function of RH for ZnO and ZnO/AuNPs in a range from 11% to 95% RH. All the ZnO/AuNPs sensors exhibit much higher response than that of ZnO sensors. The highest sensitivity is about three orders higher than that of pure ZnO when the amount of hybrid AuNPs is 6 mL, and it decreases when the amount of hybrid AuNPs increases further. A steep decrease in impedance of ZnO/AuNPs nanorods (Sample 3) is observed when the RH increases in the range from 11% to 75% RH, but the decrease in impedance is not obvious when the RH increases in the range from 75% to 95% RH. This is due to the adsorptive process of water molecules on the surface of humidity materials consists of chemisorption and physisorption. At low relative humidity, water molecules can be firstly chemisorbed on the surface of ZnO/AuNPs, and hydroxyl groups can be formed on the surface. After the chemisorbed water being formed, the amount of physically adsorbed water molecules increases with the increase of relative humidity. This physisorption water layer is located above the chemisorption layer with the increase of adsorbed water molecules [12]. At high relative humidity, there is more water molecules physically adsorbed on the surface of ZnO/AuNPs nanocomposites. This results in extra electrolyte conduction for the bridged lateral ZnO/AuNPs, which leads to a further monotonic increase in sensor conductance, so the decrease in impedance is not obvious when RH increases in the range from 75% to 95% RH, which is due to the water absorption capacity of the physisorption layer being weaker than that of the chemisorption layer. In the above processes, AuNPs play a very important role. The band gap of black ZnO with 3.07 eV can be effectively narrowed by the amount of AuNPs, which offers unique physicochemical and electronic properties for humidity sensors. Moreover, the high uniformity and dense arrays with size reduction of AuNPs hybrid ZnO can probably lead to the adsorption of a large amount of water molecules on the surface of ZnO nanocomposites. As an active component, it is worth noting that the use of AuNPs can also improve humidity sensor performance due to the catalytic effect of noble metals.

The response of a humidity sensor is strongly dependent on the test frequency. In Figure 6b, the humidity response as a function of frequency in the range from 50 Hz to 100 kHz was measured at different RH. The impedance of the ZnO/AuNPs humidity sensor decreases as frequency increases, which is due to the fact that water cannot be polarized at high frequency. In the following humidity experiments, the most suitable frequency is determined to be 100 Hz for measuring the properties of the ZnO/AuNPs (Sample 3) because of the high response and good linearity under 100 Hz.

**Figure 7:**
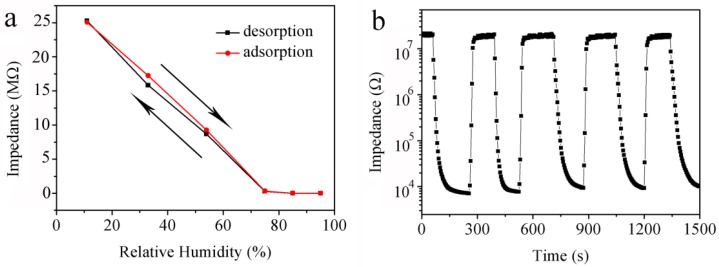
(**a**) Humidity hysteresis characteristics of ZnO/AuNPs nanorods (Sample 3) humidity sensor; (**b**) Response and recovery properties of ZnO/AuNPs nanorods (Sample 3) humidity sensor measured from 11% to 95% RH at 100 Hz.

How to minimize the hysteresis effect is crucial for a humidity sensor. If the sensing curves for the adsorption and desorption processes cover each another well, a humidity sensor will display a good reversible performance. Figure 7a shows the humidity hysteresis properties of ZnO/AuNPs humidity sensor in the processes of both adsorption and desorption in the range from 11% to 95% RH. A narrow humidity hysteresis loop shows good reversible sensing properties.

Figure 7b shows the response and recovery curves of ZnO/AuNPs humidity sensor measured by repeatedly exposing the ZnO/AuNPs sensor to 95%RH and 11% RH for four cycles at 100 Hz. Nearly identical curves over the four cycles can be observed in the response plots, which indicates the fabricated humidity sensor is highly stable. According to the literature, the response time is defined as the time taken by a sensor to achieve 90% of overall impedance change in the case of adsorption process or the recovery time in the case of adsorption and desorption process, respectively [19]. For the ZnO/AuNPs humidity sensor, the response time for the change from 11% to 95% RH is about 5.6 s, and the recovery time for the change from 95% to 11% RH is about 32.4 s. The result demonstrates that the humidity sensor with ZnO/AuNPs nanorods (Sample 3) is reversible, with a fast response, good repeatability and stable.

Experimental data showed that the highest sensitivity of ZnO/AuNPs humidity sensor is about three orders for RH levels from 11% to 95% and the response/recovery time is about 5.6 s/32.4 s, which is comparable to that of other reported favorable humidity sensors based on ZnO materials, such as ZnO with dandelion-like nanostructures [6] and laterally grown ZnO nanosheets [12].

**Figure 8:**
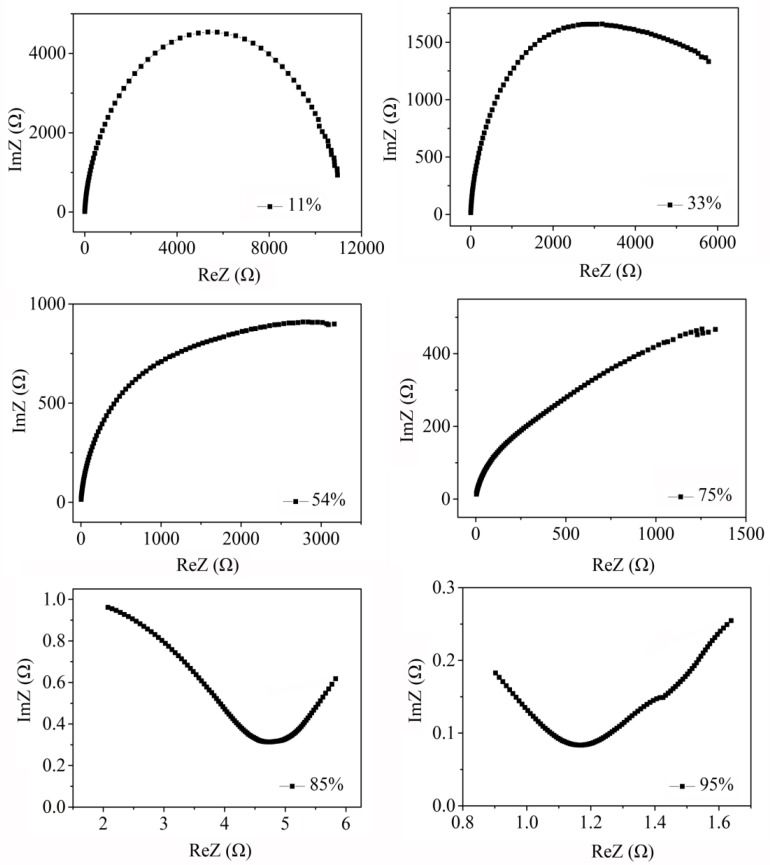
The complex impedance curve of ZnO/AuNPs (6 mL) humidity sensor measured at different RH%.

To understand the humidity sensing mechanism, the complex impedance properties of ZnO/AuNPs were measured at different RH% and they are presented in Figure 8. A small portion of semicircle to intrinsic impedance is observed at low RH% (11% and 33%), which is diminished as RH increases. At low RH%, H-bonding causes the water molecules to be chemisorbed on the surface of ZnO between oxygen atom of water molecule and hydroxyl layer. The conduction is caused by H_3_O^+^ in the region of low RH% [2,14]. Starting from 54% RH to 75% RH, there is an evident straight line in the low frequency region. As the RH increases, more water molecules are adsorbed on the surface and tend to form a liquid-like layer. Under this process, the proton conductivity plays the leading role, resulting in the discontinuity of the adsorbed water molecules [2,25]. When RH is increased to high RH (85% and 95%), the proton conductivity is changed into the ion transfer, and the free ion and water molecules are penetrated into the ZnO/AuNPs sensing film, which leads to the rapid decrease of impedance and the large increase of the capacitance for the sensors [25].

## 4. Conclusions

In summary, a highly sensitive and self-powered humidity sensor has been realized based on AuNPs hybrid black ZnO nanorods manufactured through a sol-gel method. The XRD patterns reveal that both ZnO and ZnO/AuNPs exhibit a wurtzite structure. SEM measurements show an irregular disk-like morphology. The size of pure ZnO in diameter is distributed from 60 nm to 200 nm, whereas for ZnO/AuNPs (6 mL) nanorods, high uniformity and dense arrays are obtained with a smaller diameter from 45 nm to 60 nm, which is due to the controlled ZnO nanorods growth induced by the AuNPs hybrids. EDS and TEM analysis prove the AuNPs hybrid ZnO is formed very well. Absorption spectra reveal that all the ZnO/AuNPs and pure black ZnO show a lower band gap energy compared to 3.34 eV of the typical pure ZnO. Furthermore, the band gap of ZnO/AuNPs nanocomposites is effectively influenced by the amount of AuNPs. The humidity sensing tests clearly prove that the ZnO/AuNPs nanorods exhibit much more rapid response than that of ZnO sensors and the sensitivity of such ZnO/AuNPs achieves a change three orders higher than that of pure ZnO. The response and recovery times of the ZnO/AuNPs are also improved to 5.6 s and 32.4 s, respectively. This improvement results from the uniformity and dense arrays with a smaller diameter of AuNPs hybrid ZnO. As a result, humidity adsorption on the surface of ZnO nanorods is enhanced. This study of preparing semiconductor/noble metal hybrids provides a rational way to control the morphology of semiconductor nanomaterials and to design humidity sensors with high performance.

## Figures and Tables

**Table 1:** Configuration of precursor solvents to ZnO/AuNPs.

	Sample 1	Sample 2	Sample 3	Sample 4	Sample 5
DI (mL)	10	8	4	2	0
AuNPs (mL)	0	2	6	8	10
Ethanol (mL)	20	20	20	20	20
